# Syndecan-4 is More Sensitive in Detecting Hypertensive Left Ventricular Diastolic Dysfunction in 2K2C Rats

**DOI:** 10.1155/2022/1447425

**Published:** 2022-09-24

**Authors:** Wenyue Dai, Yanqiu Liu, Fengjuan Yao, Wei Li, Jia Liu, Cuiling Li, Donghong Liu

**Affiliations:** ^1^Department of Medical Ultrasonics, the First Affiliated Hospital, Sun Yat-Sen University, Guangzhou 510080, China; ^2^Department of Medical Ultrasonics, The Eighth Affiliated Hospital of Sun Yat-Sen University, Shenzhen 518033, China

## Abstract

**Objective:**

The aim of this study was to investigate the changes of syndecan-4 (SDC-4) during the hypertensive period in two kidney-two clip (2K2C) hypertension rats and compare them to brain natriuretic peptide (BNP) and the echocardiographic parameters for diastolic function evaluation in the rat model of 2K2C hypertension.

**Methods:**

A total of 36 Sprague–Dawley (SD) rats were used in this study. Hypertension was induced in 21 by 2K2C surgery, and 15 were sham-operated. Both the 2K2C hypertension group (*n* = 21) and the sham-operated group (*n* = 15) were equally divided into 3 subgroups according to the schedules (week 4, week 8, and week 12). Serum SDC-4 and BNP were detected by ELISA, and echocardiography indexes were acquired.

**Results:**

The level of SDC-4 and cardiac fibrosis increased gradually as the experiment was processed, and BNP, Tei index, and E/E′ followed to be raised as high blood pressure was maintained after four weeks in the 2K2C hypertension rats. In the earlier 4 weeks, only SDC-4 and cardiac fibrosis were significantly increased in 2K2C hypertensive rats in comparison with normotensive rats. And it was shown that SDC-4 was positively correlated with BNP level during the entire study (*r* = 0.762, *p* < 0.01).

**Conclusion:**

SDC-4 increases gradually during the process of diastolic dysfunction in 2K2C hypertensive rats. SDC-4 is the earliest biomarker reflecting diastolic dysfunction in this model, superior to E/E′ and the Tei index. Our results indicate that serum SDC-4 could act as an early biomarker to show diastolic dysfunction.

## 1. Introduction

Hypertension is a major cause of heart failure by the initiation of left ventricular hypertrophy and coronary atherosclerosis. Left ventricular hypertrophy is not only the result of cardiac myocyte hypertrophy but also of increased extracellular matrix and collagen structure [1, 2]. Both left ventricular hypertrophy and cardiac fibroblasts lead to incomplete ventricular relaxation and progressive diastolic dysfunction [3, 4]. Cardiac fibroblasts play a key role in leading to myocardial stiffening, which could eventually result in left ventricular diastolic dysfunction. Under persistent pressure overload, cardiac fibroblasts can be activated and start to produce excessive amounts of extracellular matrix proteins [5].

Previous studies have shown that left ventricular transmembrane proteoglycan syndecan-4 (SDC-4) is increased in mice and humans through inflammatory mediators such as TNF-a and IL-1*β* after pressure overload [6, 7]. And SDC-4 promoted the differentiation of cardiac fibroblasts and the production of collagen through its dual role in collagen cross-linking in the pressure-overloaded heart [5].

Echocardiography can detect the cardiac structure and function of patients with hypertension as well as provide prognostic information. Echocardiography is a preferred method for differentiation of normal ejection with impaired diastolic function.

SDC-4 was described as essential to developing heart failure in response to pressure overload [8]. But few studies are available to explain the role of SDC-4 in diastolic dysfunction under the condition of hypertension. The aim of this study was to investigate the changes of SDC-4 during the hypertensive period in two kidney-two clip hypertension rats and to compare them with BNP and the echocardiographic data in the evaluation of diastolic function.

## 2. Materials and Methods

### 2.1. Experimental Protocol

A total of 36 healthy Sprague–Dawley (SD) male rats (weight 250–350 g, age 60–90 days) were used in the study. The rats were housed in groups of five a cage with free access to food and water, kept in consistent condition, in a temperature-controlled environment (20–25°C) with a 12 h light/dark cycle. The rats were randomly divided into 2 groups: the 2K2C hypertension group (2K2C, *n* = 21) and the sham-operated group (Sham, *n* = 15). Both the 2K2C and Sham groups were equally further divided into 3 subgroups (7 per subgroup for 2K2C and 5 for Sham). Blood pressure, weight, echocardiographic scans, myocardial fibrosis, as well as SDC-4, and BNP were recorded on 4th, 8th, and 12th week after the operation.

All experimental procedures and investigations were performed in accordance with the Guide for the Care and Use of Laboratory Animals published by the National Institutes of Health (National Institutes of Health publication, 8th Edition, 2011) and approved by the Institutional Animal Care and Use Committee and the Ethics Committee of the First Affiliated Hospital at Sun Yat-sen University.

### 2.2. Hypertension Model

The two kidney-two clip (2K2C) hypertension rat model was prepared as follows: Under anesthesia with 3% sodium pentobarbital (36 mg/kg body weight IP), a median longitudinal incision on abdominal skin was made, then both the right and left renal arteries were constricted by placing ring-shaped silver clips with an inner diameter of 0.30 mm to induce hypertension. The ring of the clip was placed around the renal artery, and then the outer gap of the clip was shut. Abdominal contents were undamaged and were carefully returned to the original location. The abdominal wall and skin were sutured. The sham-operated group also underwent the same surgical procedure but without a vascular clip [9].

### 2.3. Systolic Blood Pressure (SBP)

The SBP of every unanesthetized rat was taken in each group at 4 w, 8 w, and 12 w postoperation using the tail-cuff method. The SBP was taken three times; the average reading was recorded. Before taking the blood pressure, the rats were warmed up at 35°C in a cage for 10 minutes. This procedure was required in order to obtain constant and stable blood pressure.

### 2.4. Echocardiography

The cardiac function was evaluated by serial transthoracic echocardiography at 4 w, 8 w, and 12 w postoperation. Echocardiographic studies were performed with an 11.5-MHz transducer using a GE Vivid i digital ultrasound system (GE Healthcare, US). All scans were averaged over 3 cardiac cycles. The images were stored in DICOM format for further analysis.

A 2D guided M-mode echocardiography was obtained from the parasternal short-axis view at the papillary muscle level. The measurements of the end-diastolic ventricular septum (IVSd), end-diastolic and end-systolic left ventricular inner diameter (LVDd, LVDs), and end-diastolic left ventricular posterior wall thickness (LVPWd) were measured in this image. Then, the ejection fraction (EF) and shortening fraction (FS) were calculated by the Teich method. LA anteroposterior diameter (LAd) was measured in the parasternal long-axis view using M-mode echocardiography.

The Devereux's formula used to estimate left ventricular mass index (LVMI) was as follows:

(1) $$\begin{array}{c} \text{LVMI}=\frac{\text{LVM}}{\text{BSA}}, \end{array}$$

where LVM (left ventricular mass) was calculated as follows:

(2) $$\begin{array}{c} \text{LVM}=0.8\times 1.04\times \left[\left(\text{IVSd}+\text{LVDd}+\text{LVPWd}\right)3-\text{LVDd}3\right]+0.6. \end{array}$$

The skin surface areas (BSA) of the rats were measured using the Meeh–Rubner [10] equation.

(3) $$\begin{array}{c} \text{BSA}=K\times \left(\frac{W^{2/3}}{10000}\right), \end{array}$$

where, constant *K* is 9.1 for rats, and *W* is the body weight (g).

Mitral inflow was assessed from the apical 4-chamber view with pulsed-wave Doppler by placing a 0.5 mm sample volume between the tips of the mitral leaflets during diastole at a speed of 200 mm/s. From the mitral inflow profile, peak early diastolic left ventricular filling velocity (*E*) was measured. Tissue Doppler imaging was performed to detect *E*′ velocity by the same sample volume and speed at the septal and lateral mitral annulus. The ratio of *E* to *E*′ (*E*/*E*′) was calculated.

Left ventricular Tei is an index that estimates global systolic and diastolic ventricular function by systolic and diastolic time intervals. Pulsed-wave tissue Doppler was carried out to detect the time from mitral valve closure to opening (MCOT, ms) and left ventricular ejection time (LVET, ms). Tei index was calculated as follows: [11]

(4) $$\begin{array}{c} \frac{\text{MCOT}-\text{LVET}}{\text{LVET}}. \end{array}$$

### 2.5. Syndecan-4 and BNP

The collected samples of blood were placed in two serum separator tubes to clot for two hours at room temperature, and then they were centrifuged for 20 minutes at approximately 1000 rpm. Next, the samples were kept at −80°C until the time for tests. The titers of syndecan-4 and BNP were determined by the Sandwich Enzyme-Linked Immunosorbent Assay (ELISA), and the ELISA kits were supplied by Cloud-clone Company (Shanghai, China).

### 2.6. Histologic Analysis

Hearts were excised for histological analysis at predetermined time points. Heart samples were fixed by perfusion with 10% formaldehyde and excised immediately. Next, hematoxylin-eosin staining and Masson's trichrome staining were performed. The expression of fibrosis was quantified by computer graphic software (Image Pro Plus 6.0, Media cybernetics, America) [12].

### 2.7. Statistical Analysis

SPSS Statistics 19.0 (IBM, USA) was used for statistical analysis. The data were assessed at 4 w, 8 w, and 12 w postoperatively (*n* = 7 for 2K2C for each time point) and compared with age-matched control groups (*n* = 5 for Sham for each time point). All variables were expressed as the mean ± standard deviation. The student's independent *t*-test was used for intergroup comparisons (two groups), including SBP, echocardiographic parameters (including LVMI, EF, peak E, E/E′ and Tei index), serum BNP, and serum SDC-4. Differences between the two groups (2K2C and Sham) were considered statistically significant if *P* < 0.05. Pearson's linear correlation analysis was used to determine the correlations between serum levels of SDC-4 and BNP, CVF, *E*/*E*′, and Tei index.

Values of *P* < 0.05 were considered statistically significant.

## 3. Results

### 3.1. Weight and SBP and Serum BNP among Groups

**Figure 1:**
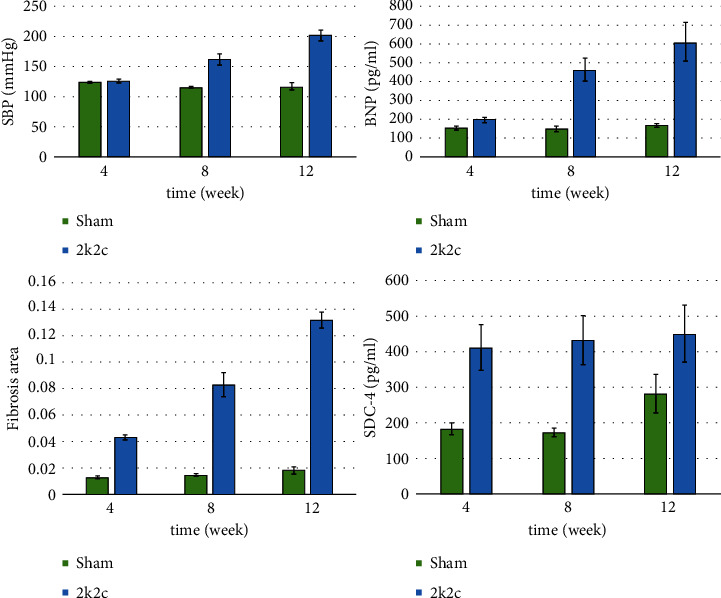
The change of Tei, BNP, SDC-4, and fibrosis area. Tei, the average of Teisep and Teilat; BNP, the level of B-type natriuretic peptide; SDC-4, the level of syndecan-4; and fibrosis area, the expression of fibrosis. Data are presented as the mean± standard deviations from each group. ^*∗*^is *P* < 0.05.

With the same feeding pattern, there were no significant differences in weight gain between the sham and 2K2C groups as well as body surface area (Table 1). The systolic blood pressure (SBP) was measured at predetermined time points (4 w, 8 w, and 12 w). There was no mortality in either the sham or 2K2C groups. The systolic blood pressure (SBP) and serum BNP in the 2K2C group were significantly higher than those in the sham group (*p* < 0.05) (shown in Table 1 and Figure 1(b)).

### 3.2. Echocardiographic Measurements

**Figure 2:**
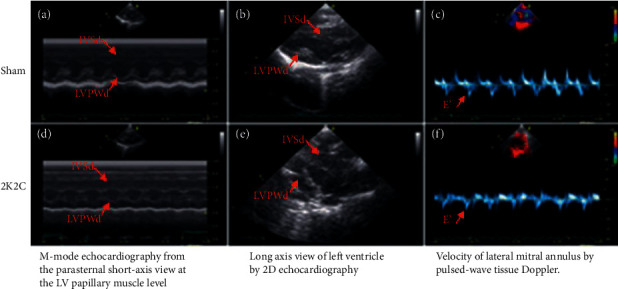
The imaging of echocardiography of the two groups. The diameter of interventricular septum and posterior wall at end-diastole (IVSd, LVPWd) were thicker in 2K2C group (d, e) than the sham group (a, b). The Velocity of mitral annulus at the early diastole (e') was lower than in 2K2C group (f) than the sham group (c).

There were no significant differences in LAd, LVDd, LVDs, EF, FS, and peak E between the sham and 2K2C groups (Table 1) (Figure 2). Compared with the sham group, IVSd, LVPWd, LVM, and LVMI significantly increased in the 2K2C hypertension group at 8 w and 12 w (*p* < 0.01) (shown in Table 1).

Differing from the sham group, Tei index and *E*/*E*′ both on the septum and lateral sides increased along with the process of hypertension in the 2K2C group (*p* < 0.01) (shown in Table 2 and Figure 1(a)).

### 3.3. Histological Analysis Showed Myocardial Fibrosis in 2K2C Group

**Figure 3:**
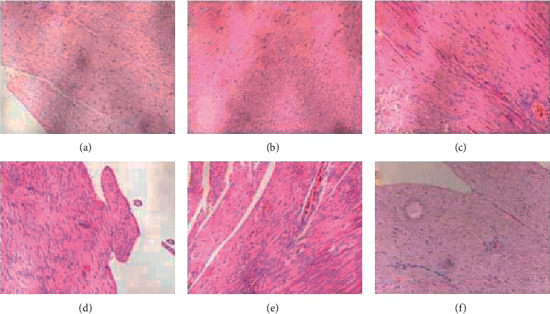
HE staining of left ventricular myocardium of two groups (×100) sham: sham-operated group; 2K2C: 2K2C hypertension group.

In the 2K2C group, myofibers were disorganized and thickened. Cardiomyocyte edema and interstitial hyperemia occurred. In the cytoplasm, it was found that newly generated capillaries appeared. While in the Sham group, the morphology, cytoplasm, and interstitium of cardiomyocytes were in a regular arrangement, and there were also myofibers with plenty of cytoplasm but few nascent capillaries (Figure 3).

**Figure 4:**
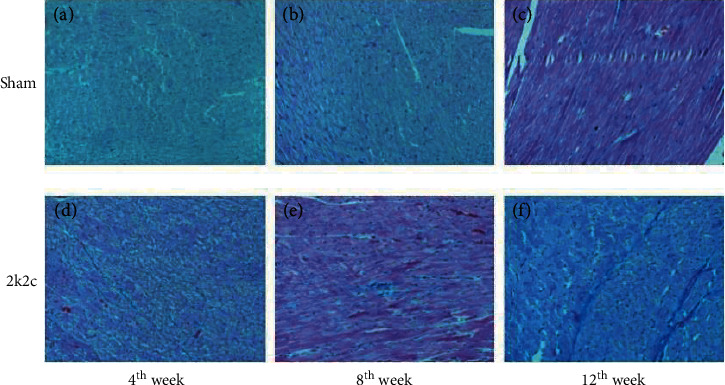
Masson staining of left ventricular myocardium of two groups (×200) sham: sham-operated group; 2K2C: 2K2C hypertension group collagenous fibers were in blue, while myocardial fibers were in red.

In the Masson staining of the left ventricular myocardium, collagenous fibers were in blue, while myocardial fibers were in red. The images of histopathological slices of the 2K2C group showed much more collagenous fibers compared with the Sham group (Figure 4). The mean of 5 fields per section was evaluated for each rat and analyzed by Image Pro Plus 6.0 and SPSS 19.0. The results revealed that there was obvious fibrotic tissue proliferation in the 2K2C group compared with the Sham group (Figure 1(c)).

### 3.4. SDC-4 with Left Ventricular Diastolic Function

The results of SDC-4 increased along with the process of hypertension (*P* < 0.05) as shown in Figure 1(d). SDC-4 presented with good correlations with *E*/*E*′ ratio, BNP, and Tei index in Pearson's analysis (*r* = 0.606, 0.762, and 0.837 respectively, *P* < 0.01), as well as with fibrosis area (CVF) (*r* = 0.721, *P* < 0.01) (Table 3). These data indicate that syndecan-4 is positively correlated with cardiac dysfunction after pressure overload in mice.

## 4. Discussion

In this study, it was found that the expression of SDC-4 was closely related to the diastolic dysfunction in the two kidney-two clip (2K2C) hypertensive rat model. As high blood pressure was maintained after four weeks in the 2K2C hypertension rats, the levels of SDC-4, cardiac fibrosis, brain natriuretic peptide (BNP), Tei index, and *E*/*E*′ were continuously increasing, which were correlated to left ventricular diastolic dysfunction.

Hypertension leads to left ventricular hypertrophy by increasing stress on the cardiac wall. But, left ventricular hypertrophy is hardly seen in early and mild hypertension; usually, diastolic dysfunction is the first sign of hypertension [13]. Echocardiography and B-type natriuretic peptides (BNPs) provide an effective assessment of cardiac function. Echocardiography is the most useful method for the noninvasive evaluation of left ventricular systolic and diastolic functions. It is proved that *E*/*E*′ ratio can be used to predict LV filling pressure, and it is an important and recommended variable for identifying left ventricular diastolic dysfunction in hypertensive patients [14].

In this study, it was found that the left atrium of hypertensive rats without treatment for 4 weeks did not enlarge, while left ventricular wall thickness and left ventricular mass obviously increased. Meanwhile, Doppler variables (including decreased *E*′ in bilateral mitral valve annulus, increased Tei index, and *E*/*E*′) had significantly changed in the hypertensive rats compared to the sham group. It was shown in this study that the left ventricular diastolic dysfunction had emerged without left atrial enlargement in the 2K2C hypertensive rats after 4 weeks continuously under high pressure.

Testing of natriuretic peptides is useful in the evaluation of patients who are suspected of having heart failure with preserved ejection fraction. When the BNP is 35 pg/ml or higher, TTE should be recommended to evaluate the left ventricle systolic and diastolic function in patients [15]. The prognosis is worsened by higher levels of brain natriuretic peptide and decreased diastolic function. In the previous study, it was shown that NT-pro BNP levels increased due to diastolic dysfunction in hypertensive patients [16, 17]. In our study, the BNP levels were raised after four weeks consistently in the hypertension group compared to the sham group in rats. The BNP level continued to elevate consistently in hypertensive rats until the end of the study. And our results shew that raised BNP level was highly correlated with increased SDC-4 during the entire study (*r* = 0.762, *P* < 0.01).

The Tei index, a Doppler parameter, is defined as the sum of the isovolumic contraction and relaxation times divided by the ejection time. It can be used to access the global left ventricular function [18]. And, the Tei index is obviously higher in patients with grade-I diastolic dysfunction with preserved ejection fraction [19].

In this study, compared to the normotensive rats, the Tei index increased gradually after four weeks in 2K2C hypertensive rats. This phenomenon is seen in most studies [20, 21]. But, in a hypertensive heart failure rat model, Shingu Y et al. reported that the Tei index was normal in diastolic dysfunction with preserved and reduced left ventricular ejection fraction [22]. However, Shingu Y et al. used a method of conventional Doppler to record left ventricular inflow and outflow for myocardial performance indicators. In our study, the Tei index accessed by conventional Doppler needs to be measured in both two different spectrums to get isovolumic diastolic and systolic time and ejection time in comparison with pulsed-wave tissue Doppler imaging (PW-TDI), which allows these time parameters to be measured in the same cardiac cycle. In addition, as stated by Shingu et al., the model used in their study might have severe diastolic dysfunction (normal IRT, prolonged ICT and ET, higher *E*/*E*′, and larger atrial), which indicated that pressure-overload elevation might contribute to the normal Tei index.

Previous studies showed that SDC-4 played an important role in compensating for hypertrophy and maintaining cardiac function during the process of heart failure due to pressure overload [23]. SDC-4 is a biomarker of left ventricular remodeling. Stress-induced SDC-4 signaling contributes to increased left ventricular myocardial stiffness [5, 24].

Pressure overload, i.e., hypertension, leads to ventricular stiffness, which may cause diastolic dysfunction. Cardiac fibroblasts play an important role in myocardial stiffness. Collagen cross-linking is one of the factors that explains the overexpressed myocardial stiffness [25–27]. The transmembrane proteoglycan SDC-4 exerts a dual role in collagen cross-linking and determines passive tension in the myocardium of pressure-overloaded hearts [5].

It is demonstrated that SDC-4 promotes myofibroblast differentiation and collagen production upon mechanical stress by the action of the nuclear factor of activated T-cells (NFAT) [6, 7]. Similar to previous work, the level of cardiac SDC-4 expression increased in the 1^st^ week in transverse aortic constriction pressure-overloaded mice [8, 23]. The results of our study showed that, similarly, compared to the normotensive rats, SDC-4 began to quickly increase in the first 4 weeks in 2K2C hypertensive rats. Additionally, after 4 weeks as the high pressure existed in 2K2C hypertensive rats, SDC-4 and left ventricle cardiac fibrosis continued to increase and it correlated with the elevated BNP, *E*/*E*′, and Tei index.

Interestingly, our study found that, in the early 4 weeks, only SDC-4 showed significant elevation in 2K2C hypertensive rats, while cardiac fibrosis increased at the same time. It demonstrated that SDC-4 was closely related to cardiac fibrosis in the overpressure-loaded model. In addition, SDC-4 might act as an early biomarker for identifying heart failure, especially in the left ventricular diastolic dysfunction in 2K2C hypertensive rats.

### 4.1. Limitation

This article is not intended to discuss the clinical situation because the hypertensive model is unable to represent the clinical status. The pathogenesis is explored in this study partially because other studies have reported some theories to explain why the expression of syndecan-4 is related to diastolic dysfunction. This study focuses on the correlation between the expression of syndecan-4 and diastolic dysfunction in hypertensive 2K2C rats, and further experimental studies are required to investigate the therapeutic effects on the changes of syndecan-4, *E*/*E*′, and Tei index.

## 5. Conclusion

The present study indicates that SDC-4 is an early biomarker that changes significantly in comparison with other data, as SDC-4 level increases gradually during the process of diastolic dysfunction in two kidney-two clip hypertensive rats. It could be implied that SDC-4 might be used as an earlier biomarker to indicate early diastolic dysfunction than echocardiography.

## Figures and Tables

**Table 1:** General Clinic and Echocardiographic Findings in rats.

Time (week)	4 w		8 w		12 w	
	Sham (*n* = 5)	2K2C (*n* = 7)	Sham (*n* = 5)	2K2C (*n* = 7)	Sham (*n* = 5)	2K2C (*n* = 7)
SBP (mmHg)	122.4 ± 2.3	126.3 ± 3.5	117.7 ± 3.2	161.7 ± 6.2^*∗*^	118.9 ± 3.0	201.8 ± 9.2^*∗*^
Weight (g)	335 ± 21	360 ± 46	440 ± 14	439 ± 15	521 ± 17	518 ± 17
BSA (m^2^)	0.044 ± 0.002	0.046 ± 0.004	0.053 ± 0.001	0.053 ± 0.001	0.059 ± 0.001	0.059 ± 0.001
LAd (mm)	2.87 ± 0.2	2.62 ± 0.3	3.38 ± 0.2	3.14 ± 0.4	3.43 ± 0.2	3.27 ± 0.3
IVSd (mm)	1.45 ± 0.1	1.73 ± 0.1	1.50 ± 0.0	1.99 ± 0.1^#^	1.48 ± 0.0	2.50 ± 0.1^#^
LVDd (mm)	5.95 ± 0.2	5.93 ± 0.4	6.90 ± 0.5	6.37 ± 0.3	6.78 ± 0.4	6.68 ± 0.8
LVDs (mm)	3.45 ± 0.2	3.93 ± 0.4	4.30 ± 0.7	3.97 ± 0.3	4.31 ± 0.3	4.25 ± 0.5
LVPWd (mm)	1.40 ± 0.0	1.63 ± 0.1	1.45 ± 0.1	1.91 ± 0.0^#^	1.46 ± 0.0	2.32 ± 0.2^#^
LVM (mg)	392.87 ± 35.9	498.10 ± 69.8	522.66 ± 52.6	687.67 ± 50.6^#^	504.68 ± 48.8	1033.97 ± 265.8^#^
LVMI (g/m^2^)	8.9 ± 0.4	10.9 ± 1.7	9.9 ± 1.2	13.1 ± 0.9^#^	8.6 ± 0.9	17.6 ± 4.4^#^
EF (%)	78.5 ± 2.1	68.7 ± 5.5	73.0 ± 5.7	73.9 ± 2.7	72.0 ± 3.6	71.8 ± 4.0
FS (%)	42.0 ± 2.8	33.7 ± 4.5	37.5 ± 5.0	37.9 ± 2.1	36.0 ± 3.1	36.0 ± 3.2

SBP, indicates systolic blood pressure; BSA, body surface area; LAd, left atrial diameter; IVSd, end-diastolic interventricular septum thickness; LVDd, end-diastolic left ventricular internal diameters; LVDs, end-systolic left ventricular internal diameters; LVPWd, end-diastolic left ventricular posterior wall thickness; LVM, left ventricular mass; LVMI, left ventricular mass index; EF, ejection fraction; FS, fractional shortening. Data are presented as the mean ± standard deviations from each group. ^#^is *P* < 0.01 and ^*∗*^is *P* < 0.05 vs. rats in the sham group.

**Table 2:** Doppler Measurements of *E*/*E*′ and Tei index.

Time (week)	4 w		8 w		12 w	
	Sham (*n* = 5)	2K2C (*n* = 7)	Sham (*n* = 5)	2K2C (*n* = 7)	Sham (*n* = 5)	2K2C (*n* = 7)
*E* (cm/s)	85.0 ± 9.9	91.7 ± 11.0	111.5 ± 13.4	120.4 ± 10.1	100.7 ± 6.7	103.3 ± 12.4
E_sep_′(cm/s)	7.9 ± 0.7	7.3 ± 1.2	8.1 ± 0.6	5.6 ± 1.3^#^	7.8 ± 0.9	5.1 ± 0.9^#^
E_lat_′(cm/s)	8.0 ± 2.2	6.3 ± 1.3	7.5 ± 0.8	5.1 ± 0.9^#^	7.9 ± 1.1	5.0 ± 0.6^#^
E′	7.9 ± 0.9	6.7 ± 1.1	7.9 ± 0.5	5.3 ± 0.7^#^	7.9 ± 0.9	5.1 ± 0.5^#^
E/E′	10.9 ± 5.1	15.0 ± 3.8	13.6 ± 1.5	21.8 ± 3.8^*∗*^	12.6 ± 1.3	20.1 ± 2.3^*∗*^
Tei_sep_	0.36 ± 0.0	0.54 ± 0.0	0.33 ± 0.0	0.60 ± 0.1^*∗*^	0.36 ± 0.0	0.75 ± 0.1^*∗*^
Tei_lat_	0.39 ± 0.0	0.53 ± 0.0	0.36 ± 0.0	0.59 ± 0.0^*∗*^	0.35 ± 0.0	0.76 ± 0.1^*∗*^
Tei	0.37 ± 0.0	0.54 ± 0.0	0.34 ± 0.0	0.60 ± 0.1^*∗*^	0.35 ± 0.0	0.75 ± 0.1^*∗*^

E, indicates peak early diastolic left ventricular filling velocity; E_sep_′, early diastolic E velocity at the the septum of mitral annular; E_lat_′, early diastolic E velocity at the lateral of mitral annular; E′, the average of E_sep_′ and E_lat_′; E/E′, the ratio of E to E′; Tei_sep_, Tei index at the septum of mitral annular; Tei_lat,_ Tei index at the lateral of mitral annular; Tei, the average of Tei_sep_ and Tei_lat_. Data are presented as the mean ± standard deviations from each group. ^#^is *P* < 0.01 and ^*∗*^is *P* < 0.05.

**Table 3:** Pearson's linear correlation analysis of SDC-4 level and diastolic function.

	*R*	*P*
Tei	0.837	*P* < 0.01
E/E′	0.606	*P* < 0.01
BNP	0.762	*P* < 0.01
Fibrosis area (CVF)	0.721	*P* < 0.01

## Data Availability

The datasets that support the findings of this study are available on request from the corresponding authors, Donghong Liu and Cuiling Li. The data are not available to the public due to the nature that the information could compromise the interest of research participants.

## Abbreviations

2K2C: Two kidney-two clip
SDC-4: Syndecan- 4
SBP: Systolic blood pressure
EF: Ejection fraction
LA: Left atrial
LV: Left ventricle
LVM: Left ventricular mass
LVMI: Left ventricular mass index
BNPs: B-Type natriuretic peptides.
